# Fe_70−*x*_Nd_7_B_21_Zr_2_Nb*_x_* (*x* = 0–3.0) Permanent Magnets Produced by Crystallizing Amorphous Precursors

**DOI:** 10.3390/ma17061429

**Published:** 2024-03-20

**Authors:** Yong Gu, Zili Wang, Hui Xu, Zhong Li

**Affiliations:** 1 School of Engineering & Qianjiang College, Hangzhou Normal University, Hangzhou 310018, China; 2 State Key Laboratory of Fluid Power & Mechatronic Systems, Zhejiang University, Hangzhou 310027, China; ziliwang@zju.edu.cn; 3 School of Materials Science and Engineering, Shanghai University, Shanghai 200072, China; huixu8888@shu.edu.cn; 4 College of Materials and Environmental Engineering, Hangzhou Dianzi University, Hangzhou 310018, China; hanying880205@hdu.edu.cn

**Keywords:** amorphous alloy, annealing, hard magnetic properties, remanence

## Abstract

The phase evolution, magnetic properties and microstructure of rod-shaped permanent magnets prepared by annealing the amorphous precursor Fe_70−*x*_Nd_7_B_21_Zr_2_Nb*_x_* (*x* = 0–3.0) were systematically studied. X-ray diffraction analysis, magnetometer, microstructure and δM-plots studies show that the good magnetic properties of the magnet are attributed to the uniform microstructure composed of exchange-coupled α-Fe and Nd_2_Fe_14_B phases. Nb addition to Fe_67.5_Nd_7_B_21_Zr_2_Nb_2.5_ alloy led to an increase in the volume fraction of the soft magnetic phase, reinforced exchange coupling and improved magnetic properties. The magnetic properties of the optimized annealed Fe_67.5_Nd_7_B_21_Zr_2_Nb_2.5_ rod are: coercivity (*H_ci_*) = 513.92 kA/m, remanence (*B_r_*) = 0.58 T, squareness (*H_k_/H_ci_*) = 0.24 and magnetic energy product (*(BH)_max_*) = 37.59 kJ/m^3^.

## 1. Introduction

Fe-based bulk amorphous alloys (BAA) have aroused widespread interest due to their excellent magnetic properties (MPs), mechanical properties and low raw material costs [1,2]. Usually, Fe-based BAAs exhibit soft magnetic properties (SMPs) in the as-cast state. In 2002, Zhang et al. [3] studied the crystallization process of a rod-shaped Fe_67_Co_9.4_Nd_3.1_Dy_0.5_B_20_ BAA with a diameter of 0.5 mm and found that after crystallization, the MPs of the alloy changed from soft to hard magnetic, that is, an Fe-based bulk permanent magnet alloy is obtained. This rare earth-containing Fe BAA is called ”Re-Fe-based BAA” [4]. The crystallization of such BAAs can obtain permanent magnet materials, which not only provides a new direction for the application of BAAs, but also provides a new method for preparing high-density permanent magnets.

Previous studies [5] have found that Fe_61_Nd_10_B_25_Nb_4_ alloys exhibit excellent permanent MPs after composition adjustment and optimal heat treatment, with coercivity (*H_ci_*) as high as 1191kA/m, remanence (*B_r_*) of 0.42 T, and a maximum magnetic energy product (*(BH)_max_*) of 31.72 kJ/m^3^. Zhang et al. [6] developed a rod-shaped Fe_64.32_Nd_9.6_B_22.08_Nb_4_ BAA with *H_ci_* as high as 1100 kA/m; however, *B_r_* and (*BH*)*_max_* are 0.44 T and 32.96 kJ/m^3^ after annealing for 5 min at 983 K. Subsequently, Cui et al. [7] and Man et al. [5] also investigated the Fe-Nd-B-Nb system alloys, and they successfully prepared bar-shaped Fe_71.5_Nd_9_B_15.5_Nb_4_ and flake-shaped Fe_61_Nd_10_B_25_Nb_4_ BAAs, and the alloys were crystallized with 1154 kA/m and 1191.1 kA/m *H_ci_*, and 0.59 T and 0.42 T *B_r_*. Yan et al. [8,9,10] studied the Fe-Nd-B-Mo alloy system and prepared a rod-shaped Fe_67_Nd_7_Mo_3_B_22_Zr_1_ BAA. After annealing at 1013 K for 10 min, its *B_r_* and (*BH*)*_max_* were 0.53 T and 49.52 kJ/m^3^, respectively. However, although these types of alloys can obtain high coercivity, they generally have the disadvantage of low remanence, so how can the remanence and comprehensive MPs of the alloys be improved?

This study focuses on developing high-MP NdFeB permanent magnets through suction casting and one-step annealing technology. The Fe_70−*x*_Nd_7_B_21_Zr_2_Nb*_x_* (*x* = 0–3.0) alloys are selected based on our previous works [11]. The reasons for choosing Nb elements are: (1) The addition of appropriate Nb element [12,13,14] can enhance the amorphous formation ability of certain Fe-based alloys. Because the addition of the element Nb conforms to the three empirical laws proposed by Inoue for the formation of BAAs [15,16]: (a) the alloy consists of three or more group elements; (b) the difference between the atomic sizes of Nb and the major elements in the alloys are large (Nb-Fe: 15.27%, Nb-B: 43. 62%), the addition of Nb induces a significant change in the topological parameter, leading to a more chaotic arrangement of atoms, thus increasing the viscosity and lowering the diffusion rate of the liquid alloy, which is conducive to the formation of an amorphous structure; (c) Nb and the major elements in the alloy have a relatively large negative heat of mixing (Nb-Fe: −16 kJ/mol, Nb-B: −54 kJ/mol). (2) The incorporation of suitable Nb element can contribute to the improvement of the remanence and squareness of permanent magnets [17,18,19,20,21]. (3) Additionally, some studies indicated that Nb element acts as an effective additive for refining the grain size of NdFeB magnets [22,23,24]. The effect of Nb content on phase evolution as well as MPs and microstructural properties is studied. 

The remainder of this paper is structured as follows: Section 2 summarizes the experimental procedure for sample preparation and standardization of the alloy systems. Section 3 presents the experimental results of the alloy samples. Finally, Section 4 draws the conclusions of this paper based on the experimental results.

## 2. Experimental Procedure

The WK-II (Beijing WuKe-II) vacuum arc-melting furnace was utilized to produce a master alloy with a nominal composition of Fe_70−*x*_Nd_7_B_21_Zr_2_Nb*_x_* (*x* = 0–3.0) (atomic percentage) under a high-purity argon atmosphere. The metals Fe, Nd, Zr and Nb are all high-purity (≥99.99%) metals, while B is added in form of an Fe-B alloy. To ensure the uniformity of composition of the master alloys, each ingot underwent four repeated smelting processes. The copper mold suction casting technology was employed to remelt the alloys under argon gas protection, resulting in the production of rods with a diameter of 2 mm. The density of the alloy ingots was determined using the Archimedes drainage method. Subsequently, the alloy rods were heat-treated in a quartz tube furnace with a vacuum level of 3 × 10^−3^ Pa, followed by rapid cooling after a 10 min heat preservation period. The heat treatment temperature ranged from 973 to 1023 K. The X-ray diffraction patterns (XRD-Ps) (XRD, Rigaku Corporation, Akishima-Shi, Tokyo, Japan) of the samples were measured using a D/max-2200 X-ray diffractometer manufactured, with a scanning rate of 1°/min. Thermal analysis was performed on the sample using a NETZSCH DSC 404C **(**Diamond DSC, Perkin-Elmer, New Rochelle, NY, USA) high-temperature differential scanning calorimeter. The MPs of the sample were evaluated using a Lake Shore 7407 vibrating sample magnetometer (VSM, LakeShore Cryotronics, Westerville, OH, USA), and the magnetic interaction curve of the alloy (i.e., δM-H plots) was measured using the Quantum Design PPMS-9 (PPMS-9T, Quantum Design, San Diego, CA, USA) multifunctional physical property measurement system. Microstructure was examined using a transmission electron microscope (TEM) (JEM-2100F, JEOL Ltd., Tokyo, Japan). 

## 3. Results and Discussion

### 3.1. Characteristics of As-Cast Rods

**Figure 1:**
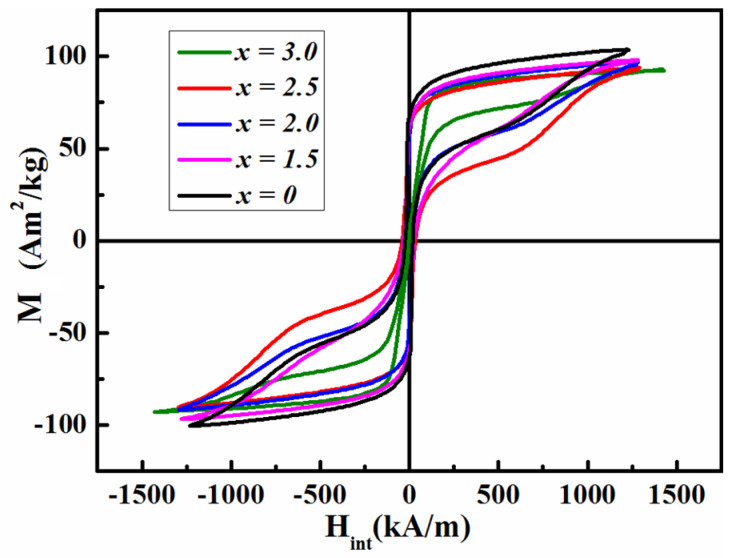
Magnetic hysteresis loops for as-cast Fe_70−*x*_Nd_7_B_21_Zr_2_Nb*_x_* (*x* = 0–3.0) alloys.

Figure 1 illustrates the magnetic hysteresis loops for the as-cast Fe_70−*x*_Nd_7_B_21_Zr_2_Nb*_x_* (*x* = 0–3.0) alloys. It can be observed that all the loops exhibit a bee waist shape. The saturation magnetization (*M_s_*) gradually decreases from 105.95 to 92.85 Am^2^/kg with the addition of the element Nb. It suggests that the inclusion of a small amount of Nb element results in a reduction in *M_s_* of the alloys. In addition, the density (ρ) also increases from 7.40 to 7.48 g/cm^3^ as the Nb content increases. The soft magnetic parameters of the as-cast samples are documented in Table 1.

**Figure 2:**
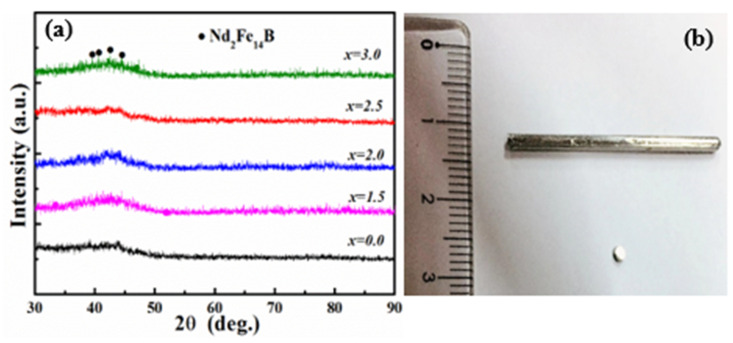
XRD-Ps for the as-cast Fe_70−*x*_Nd_7_B_21_Zr_2_Nb*_x_* (*x* = 0–3.0) samples (**a**) and the surface appearance of the as-cast *x* = 2.5 rod (**b**).

Figure 2a presents the XRD-Ps of the as-cast Fe_70−_*_x_*Nd_7_B_21_Zr_2_Nb*_x_* (*x* = 0–3.0) samples. For *x* = 0, a single broad peak is observed along with some additional peaks, suggesting the alloy contains a significant amount of amorphous phases and a small amount of crystallization phases. The XRD-Ps for *x* = 1.5, 2.0, and 2.5 alloys only has large steamed bun peaks, indicating that the alloy is basically amorphous. When *x* is further increased to 3.0, the XRD-P shows additional diffraction peaks, indicating the formation of the Nd_2_Fe_14_B phase. Figure 2b presents the surface appearance of the *x* = 2.5 rod with a diameter of 2 mm. The image illustrates a metallic cluster without any signs of surface degradation or rupture, which is a typical characteristic of a BAA. 

### 3.2. Magnetic Properties

**Figure 3:**
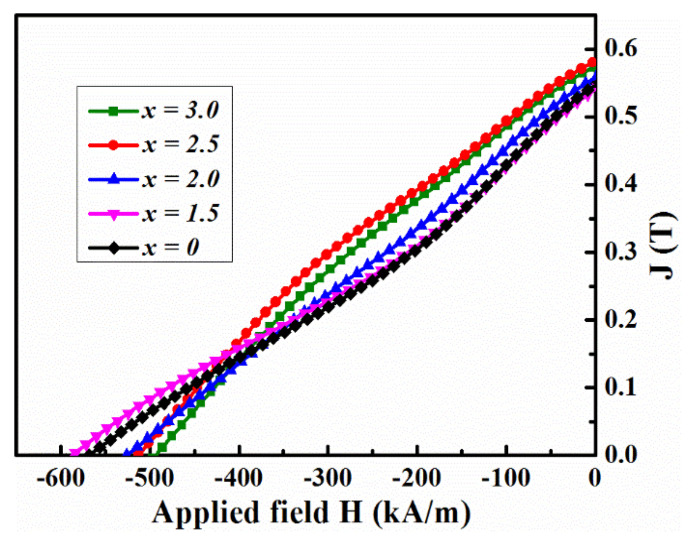
Demagnetization curves for the Fe_70−*x*_Nd_7_B_21_Zr_2_Nb*_x_* (*x* = 0–3.0) magnets annealed at *T_a_*.

**Figure 4:**
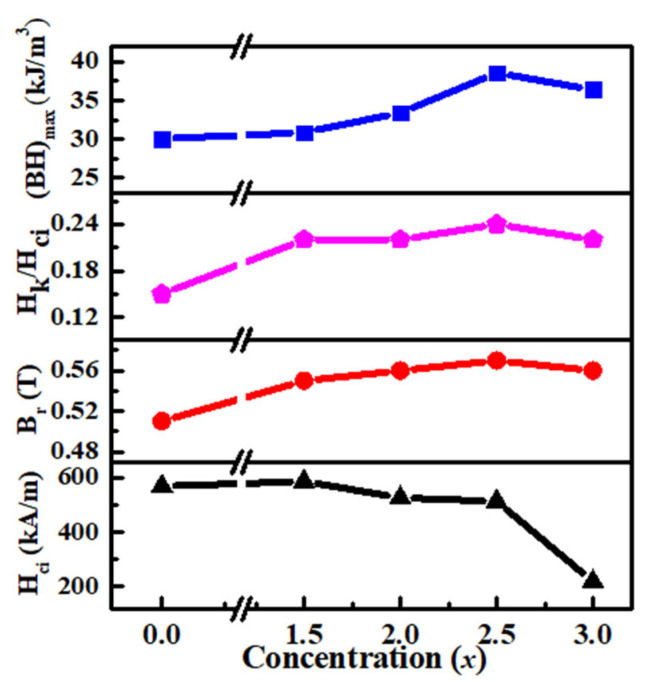
The hard magnetic parameters as a function of Nb concentration (*x*) for Fe_70−*x*_Nd_7_B_21_Zr_2._Nb*_x_*(*x* = 0–3.0) alloys.

According to the DSC results (See Supplementary Figure S1), the as-cast alloys were annealed at various temperatures (973–1023 K) for 10 min. The values of the hard magnetic properties (HMPs) for the annealed samples are recorded in Table 2. The optimum annealing temperature (*T_a_*) was determined as the temperature at which the (*BH*)*_max_* was achieved. Figure 3 shows the demagnetization curves for the alloys annealed at *T_a_*, Figure 4 illustrates the variations in *B_r_*, *H_ci_*, and squareness((*H_k_/H_ci_*), where *H_k_* is knee-point coercivity) and *(BH)_max_* is a function of *x* for the annealed Fe_70−*x*_Nd_7_B_21_Zr_2_Nb*_x_* (*x* = 0–3.0) alloys at *T_a_*. It can be observed that as *x* increases, *B_r_* gradually decreases, while *H_ci_*, *H_k_/H_ci_* and (*BH*)*_max_* initially increase and then decrease. The maximum value of *H_ci_* is obtained when *x* = 1.5. Whereas the maximum values of *H_k_/H_ci_* and (*BH*)*_max_* are achieved when *x* = 2.5. These results indicate that the addition of Nb element improves *H_ci_*, *H_k_/H_ci_* and (*BH*)*_max_* in Fe_70−*x*_Nd_7_B_21_Zr_2_Nb*_x_* (*x* = 0–3.0) alloys. The optimal HMPs of *B_r_* = 0.58 T, *H_ci_* = 513.92 kA/m, *H_k_*/*H_ci_* = 0.24, and *(BH)_max_* = 37.59 kJ/m^3^ were achieved for the *x* = 2.5 alloy.

### 3.3. XRD-Ps and Phase Compositions

**Figure 5:**
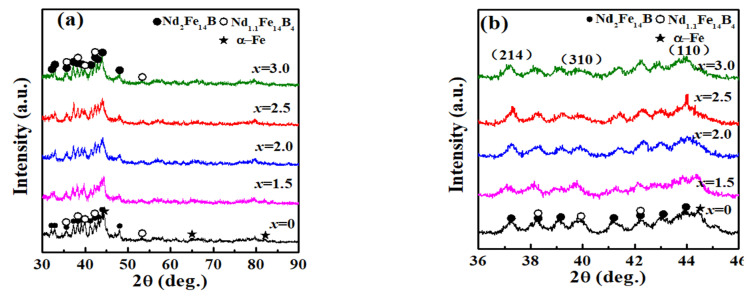
(**a**) XRD-Ps of Fe_70−*x*_Nd_7_B_21_Zr_2_Nb*_x_* (*x* = 0–3.0) magnets annealed at *T*_a_; (**b**) the enlargement of the XRD-Ps at 2θ = 36–46°.

Figure 5 shows the XRD-Ps of the Fe_70-*x*_Nd_7_B_21_Zr_2_Nb*_x_*(*x* = 0–3.0) alloys after annealing at the optimum temperature, and the relative intensity ratios of the diffraction peaks of the phases are shown in Table 3. It can be seen with the addition of the element Nb to the Fe_70−*x*_Nd_7_B_21_Zr_2_Nb*_x_* (*x* = 0–3.0) alloys, the diffraction peaks were all indexed to α-Fe, Nd_2_Fe_14_B and Nd_1.1_Fe_4_B_4_ phases. The intensities of (110) plane for α-Fe, (214) plane for Nd_2_Fe_14_B and (310) plane for Nd_1.1_Fe_4_B_4_ phase diffractions, which are used to estimate the relative volume fraction of α-Fe, Nd_2_Fe_14_B and Nd_1.1_Fe_4_B_4_ phases, are denoted as *I*_(110)Fe_, *I*_(214)2:14:1_ and *I*_(310)1.1:4:4_, respectively. As shown in Table 3, with the increase in Nb in the alloys, both the values of *I*_(110)Fe_/*I*_(214)2:14:1_ and *I*_(110)Fe_*/I*_(310)1.1:4:4_ gradually increase and then decrease, and reach the maximum value when *x* = 2.5. It indicates that the relative content of α-Fe in the alloys first increases and then decreases, that is to say, the addition of appropriate Nb is favorable to the precipitation of α-Fe, which may be the main reason leading to the *B_r_* of the alloy increase. Therefore, the presence of the Nb element plays a crucial role in adjusting the precipitation phase and enhancing the MPs. The Fe_67.5_Nd_7_B_21_Zr_2_Nb_2.5_ alloy, annealed at 993 K, demonstrated good HMPs, likely due to the strong exchange coupling interaction (ECI) between soft and hard magnetic phases (SHMPs). 

### 3.4. ECI and Microstructure

**Figure 6:**
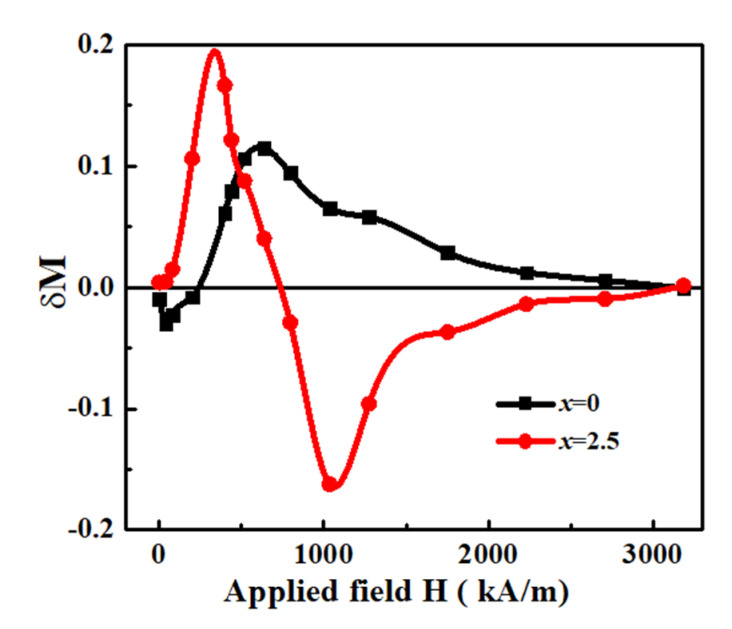
δM plots as a function of applied field for Fe_70_Nd_7_B_21_Zr_2_ and Fe_67.5_Nd_7_B_21_Zr_2_Nb_2.5_ alloys.

To understand the behavior of ECI between the SHMPs for Fe_70_Nd_7_B_21_Zr_2_ and Fe_67.5_Nd_7_B_21_Zr_2_Nb_2.5_ magnets, the δM plot [25] was constructed. It is defined as δM = [m_d_(H) − {1−2m_r_(H)}], where M_d_(H) is the reduced demagnetization remanence and M_r_(H) is the reduced magnetization remanence. δM = 0, whereas nonzero δM indicates the presence of interactions. Figure 6 depicts the δM plot as a function of the applied magnetic field for the two samples. Comparing these alloys, the Fe_67.5_Nd_7_B_21_Zr_2_Nb_2.5_ alloy displays a higher positive δM peak, suggesting a stronger ECI between the phases in comparison to the Fe_70_Nd_7_B_21_Zr_2_ alloy. This indicates that the introduction of Nb has a beneficial impact on establishing robust ECI within the magnetic phases of the Fe_67.5_Nd_7_B_21_Zr_2_Nb_2.5_ alloy. The strong ECI phenomena in the Fe_67.5_Nd_7_B_21_Zr_2_Nb_2.5_ alloy can be attributed to the fine grain size, ideal volume fractions of SHMPs, as well as their homogeneous distribution in the microstructure.

**Figure 7:**
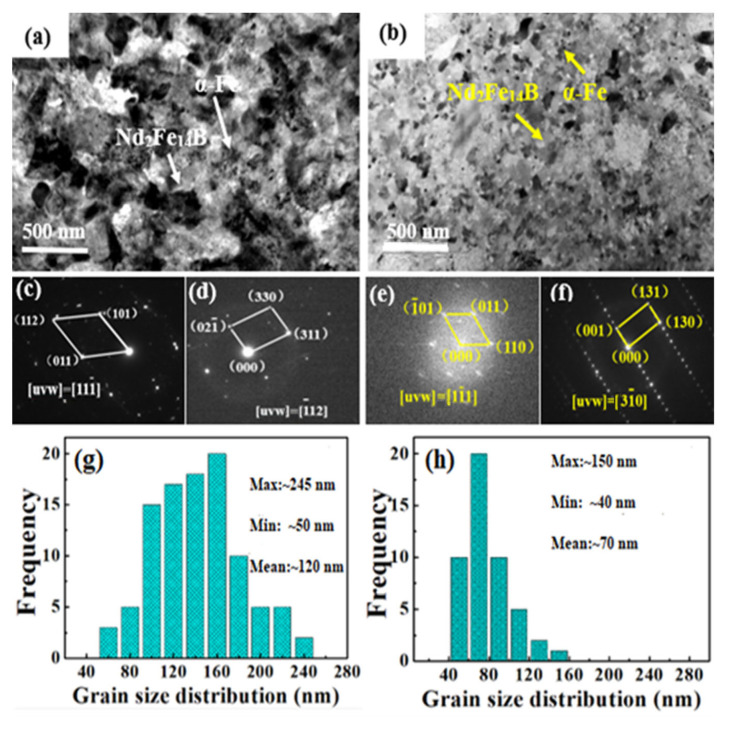
TEM micrographs of the Fe_70_Nd_7_B_21_Zr_2_ and Fe_67.5_Nd_7_B_21_Zr_2_Nb_2.5_ alloys. (**a**,**b**) Bright field images for Fe_70_Nd_7_B_21_Zr_2_ and Fe_67.5_Nd_7_B_21_Zr_2_Nb_2.5_ alloys; (**c**,**d**) selected area electron diffraction (SAED) of α-Fe and Nd_2_Fe_14_B phases for Fe_70_Nd_7_B_21_Zr_2_ alloy; (**e**,**f**) SAED of α-Fe and Nd_2_Fe_14_B phases for Fe_67.5_Nd_7_B_21_Zr_2_Nb_2.5_ alloy; (**g**,**h**) grain size distribution histograms of Fe_70_Nd_7_B_21_Zr_2_ and Fe_67.5_Nd_7_B_21_Zr_2_Nb_2.5_ alloys.

To clearly characterize the internal structure of the alloys, TEM bright field images of Fe_70_Nd_7_B_21_Zr_2_ and Fe_67.5_Nd_7_B_21_Zr_2_Nb_2.5_ alloys after optimal heat treatment are shown in Figure 7a and Figure 7b, respectively. As shown in Figure 7a, the annealed Fe_70_Nd_7_B_21_Zr_2_ sample mainly consists of α-Fe (see Figure 7c) phase and Nd_2_Fe_14_B phase (see Figure 7d). Apparently, the Fe_67.5_Nd_7_B_21_Zr_2_Nb_2.5_ sample also consists of α-Fe phase (see Figure 7e) and Nd_2_Fe_14_B phase (see Figure 7f). It is evident that the annealed Fe_70_Nd_7_B_21_Zr_2_ alloy without Nb exhibits a coarse and uneven distribution of grain sizes, with some individual grains exceeding 150 nm (see Figure 7g). Consequently, the MPs of this alloy are poor. On the other hand, the Fe_67.5_Nd_7_B_21_Zr_2_Nb_2.5_ alloy with 2.5at% Nb shows a refined and more evenly distributed grain size, with an average size of approximately 70 nm (see Figure 7h). This optimized microstructure promotes enhanced ECI between the SHMPs, resulting in improved MPs. The addition of Nb elements significantly refines the grain size of the alloy, which further enhances the ECI between the SHMPs, thereby improving the remanence.

The better MPs of Fe_67.5_Nd_7_B_21_Zr_2_Nb_2.5_ alloy compared to those of the reported α-Fe/Nd_2_Fe_14_B magnets are speculated to be due to the appropriate alloy composition, especially the Fe: Nd ratio as well as the existence of an ideal microstructure. That is to say, the higher HMPs in the alloy is ascribed to three factors: First, the formation of grains, which leads to strong magnetic exchange interactions between magnetically soft and hard phases. Second, the soft phase increment. Third, the existence of the fine grain boundary phase, which would be favorable to relate the magnetization reverse.

## 4. Conclusions

In summary, the Fe_70−*x*_Nd_7_B_21_Zr_2_Nb*_x_* (*x* = 0–3.0) bulk permanent alloys were prepared by annealing the BAAs. The MPs of the alloys changed from soft to hard magnetic, and the Fe-based bulk permanent magnet alloys are obtained. Optimal HMPs are obtained under 993 K for 10 min. HMPs are affected by the types of phases, grain size, volume fraction, and their distribution in the structure. An appropriate addition of Nb can refine the microstructure and help to enhance the ECI, thereby increasing the (BH)_max_ of the Fe_67.5_Nd_7_B_21_Zr_2_Nb_2.5_ rod magnet. The optimal HMPs, such as *B_r_* = 0.58 T and (*BH*)*_max_* = 37.59 kJ/m^3^, have been achieved, with a 2 mm diameter. The focus on the development of large-sized magnets and the characterization of MPs and microstructural parameters will greatly assist in the design of new NdFeB-based magnets suitable for scientific applications.

## Figures and Tables

**Table 1:** The soft magnetic parameters for as-cast Fe_70−*x*_Nd_7_B_21_Zr_2_Nb*_x_* (*x* = 0–3.0) alloys.

Alloys	*M_s_* (Am^2^/kg)	*H_c__i_* (kA/m)	*ρ* (g/cm^3^)
*x* = 0.0	106.60	4.65	7.40
*x* = 1.5	104.21	2.98	7.43
*x* = 2.0	97.54	8.88	7.44
*x* = 2.5	93.76	8.75	7.46
*x* = 3.0	92.85	5.32	7.48

**Table 2:** Optimum temperature *T_a_* and magnetic parameters for Fe_70−*x*_Nd_7_B_21_Zr_2_Nb*_x_* (*x* = 0–3.0) alloys.

Alloys	*T_a_* (K)	*H_c__i_*(kA/m)	*B_r_* (T)	*H_k_/H_c_* *_i_*	*(BH)_max_* (kJ/m^3^)
*x* = 0.0	1003	569.46	0.52	0.13	30.08
*x* = 1.5	973	587.42	0.53	0.19	30.87
*x* = 2.0	1003	526.66	0.56	0.22	33.43
*x* = 2.5	993	513.92	0.58	0.24	37.59
*x* = 3.0	1003	489.14	0.56	0.20	36.41

**Table 3:** Ratios of the intensity of peaks in XRD-Ps of Fe_70−*x*_Nd_7_B_21_Zr_2_Nb*_x_* (*x* = 0–3.0) magnets annealed at *T*_a._

Alloys	*I*_(110)Fe_ */I*_(214)2:14:1_	*I*_(110)Fe_ */I*_(310)1.1:4:4_	*I*_(214)2:14:1_ */I*_(310)1.1:4:4_
*x* = 0.0	1.12	2.21	1.93
*x* = 1.5	1.23	2.78	2.16
*x* = 2.0	1.32	3.46	2.63
*x* = 2.5	1.93	4.07	2.22
*x* = 3.0	1.67	3.29	1.89

## Data Availability

Data are contained within the article and Supplementary Materials.

## Supplementary Materials

The following supporting information can be downloaded at: https://www.mdpi.com/article/10.3390/ma17061429/s1, Figure S1: DSC traces for as-cast Fe_70−*x*_Nd_7_B_21_Zr_2_Nb*_x_* (*x* = 0–3.0) alloys.
