# Supplementary figures and images for: Fe70−xNd7B21Zr2Nbx (x = 0–3.0) Permanent Magnets Produced by Crystallizing Amorphous Precursors
Source: Materials (Basel). 2024 Mar 20;17(6):1429. doi: 10.3390/ma17061429 (PMC10972246; doi:10.3390/ma17061429)

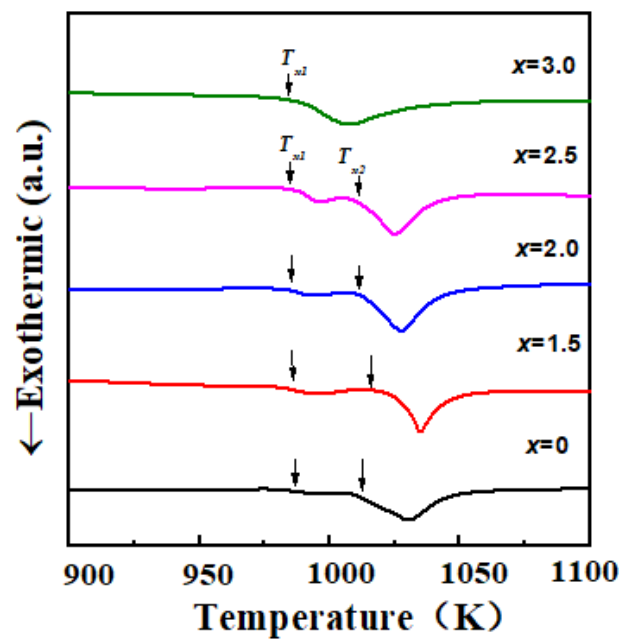

Figure S1. DSC traces for as-cast Fe<sub>70-x</sub>Nd<sub>7</sub>B<sub>21</sub>Zr<sub>2</sub>Nb<sub>x</sub> ( $x=0-3.0$ ) alloys.

Supplement: Supplementary file 1 [file materials-17-01429-s001.zip › materials-2905958-supplementary.pdf]
